# Subendocardial quantification enhances coronary artery disease detection in ^18^F-flurpiridaz PET

**DOI:** 10.1007/s00259-025-07174-6

**Published:** 2025-03-05

**Authors:** Valerie Builoff, Mark Lemley, Robert J. H. Miller, Hidesato Fujito, Giselle Ramirez, Paul Kavanagh, Christopher Buckley, Marcelo Di Carli, Daniel S. Berman, Piotr Slomka

**Affiliations:** 1 Departments of Medicine, (Division of Artificial Intelligence in Medicine), and Imaging, Cedars-Sinai Medical Center, 6500 Wilshire Boulevard, Los Angeles, CA 90048 USA; 2 Department of Cardiac Sciences, University of Calgary, Calgary, AB Canada; 3 Department of Cardiology, Nihon University Itabashi Hospital, Tokyo, Japan; 4 Pharmaceutical Diagnostics, GE Healthcare, Buckinghamshire, UK; 5 Division of Nuclear Medicine and Molecular Imaging, Department of Radiology, Division of Cardiovascular Medicine, Department of Medicine, Brigham and Women’s Hospital, Boston, MA USA

**Keywords:** ^18^F-flurpiridaz, Coronary artery disease, PET-MPI, Perfusion quantitation, Subendocardial perfusion

## Abstract

**Purpose:**

The new high resolution positron emission tomography (PET) myocardial perfusion imaging tracer, ^18^F-flurpiridaz, is set to enter clinical use soon following its recent regulatory approval. We developed an approach for evaluating subendocardial analysis for stress total perfusion deficit (TPD) and ischemic TPD, assessed its performance for detection of coronary artery disease (CAD) and compared these measures to transmural analysis and expert physician assessments.

**Methods:**

Myocardial perfusion image data from the ^18^F-flurpiridaz phase III clinical trial (NCT01347710) were used. The subendocardial layer was automatically defined on the left ventricular contours and used for the derivation of polar maps. Areas under the receiver operating characteristic curve (AUC) for quantitative and visual measures were evaluated for detecting CAD, defined as ≥ 50% stenosis by invasive coronary angiography.

**Results:**

In total, 753 cases were analyzed, with a median age of 63 (interquartile range 56,69) and 69% male. AUC for detecting ≥ 50% stenosis was higher for subendocardial than transmural analysis for stress (0.795 vs. 0.762, respectively; *p* = 0.013) and ischemic (0.795 vs. 0.767, respectively; *p* = 0.049) TPD. Subendocardial and transmural TPD achieved diagnostic performance greater than or comparable to that of the readers’ assessments in the total population as well as across subgroups of interest.

**Conclusion:**

Subendocardial analysis of ischemic perfusion improves the detection of CAD compared to transmural quantitative analysis or expert visual reading. These measures can be derived automatically with minimal user interaction. Integrating TPD quantitative measures could standardize the diagnostic approach for this novel tracer.

**Supplementary Information:**

The online version contains supplementary material available at 10.1007/s00259-025-07174-6.

## Introduction

^18^F-flurpiridaz, a new positron emission tomography (PET) myocardial perfusion imaging (MPI) tracer, has shown efficacy in the detection of coronary artery disease (CAD) and is anticipated to be adopted in routine clinical practice soon [1]. PET is the recommended perfusion imaging modality by the American Society of Nuclear Cardiology and Society of Nuclear Medicine and Molecular Imaging [2]; however, the currently available PET tracers - rubidium-82 (^82^Rb), nitrogen-13-ammonia (^13^N-ammonia), and oxygen-15-water (^15^O-water) - present logistical challenges and limitations that hinder their widespread use, primarily due to their short half-lives [3, 4]. ^18^F-flurpiridaz, without these limitations and with high image quality, has the potential to rapidly expand PET imaging. Two phase III studies have been completed for ^18^F-flurpiridaz: flurpiridaz-301 [1] and flurpiridaz-303 [5], and it has recently received US Food and Drug Administration approval for clinical use.

One of the key advantages of the F-18-based tracer is its high image resolution [3], which could extend quantitative approaches to include subendocardial imaging– a potentially sensitive marker for moderate or borderline obstructive CAD [6–9], as well as underlying pathophysiology and cardiovascular risk [8]. This represents a long-sought goal in PET imaging. However, subendocardial PET has not yet demonstrated an improvement in diagnostic performance over transmural analysis. The superior image resolution provided by an F-18-based tracer, such as ^18^F-flurpiridaz, presents a unique opportunity to explore the potential added value of subendocardial PET imaging. This post-hoc analysis of the first of two ^18^F-flurpiridaz phase III clinical trials (the flurpiridaz-301 trial) compares the diagnostic performance of subendocardial and transmural automated quantitative analysis, along with visual assessments by three expert readers, in detecting CAD, defined as stenosis ≥ 50% by quantitative invasive coronary angiography (ICA).

## Methods

### Study patients

**Fig. 1:**
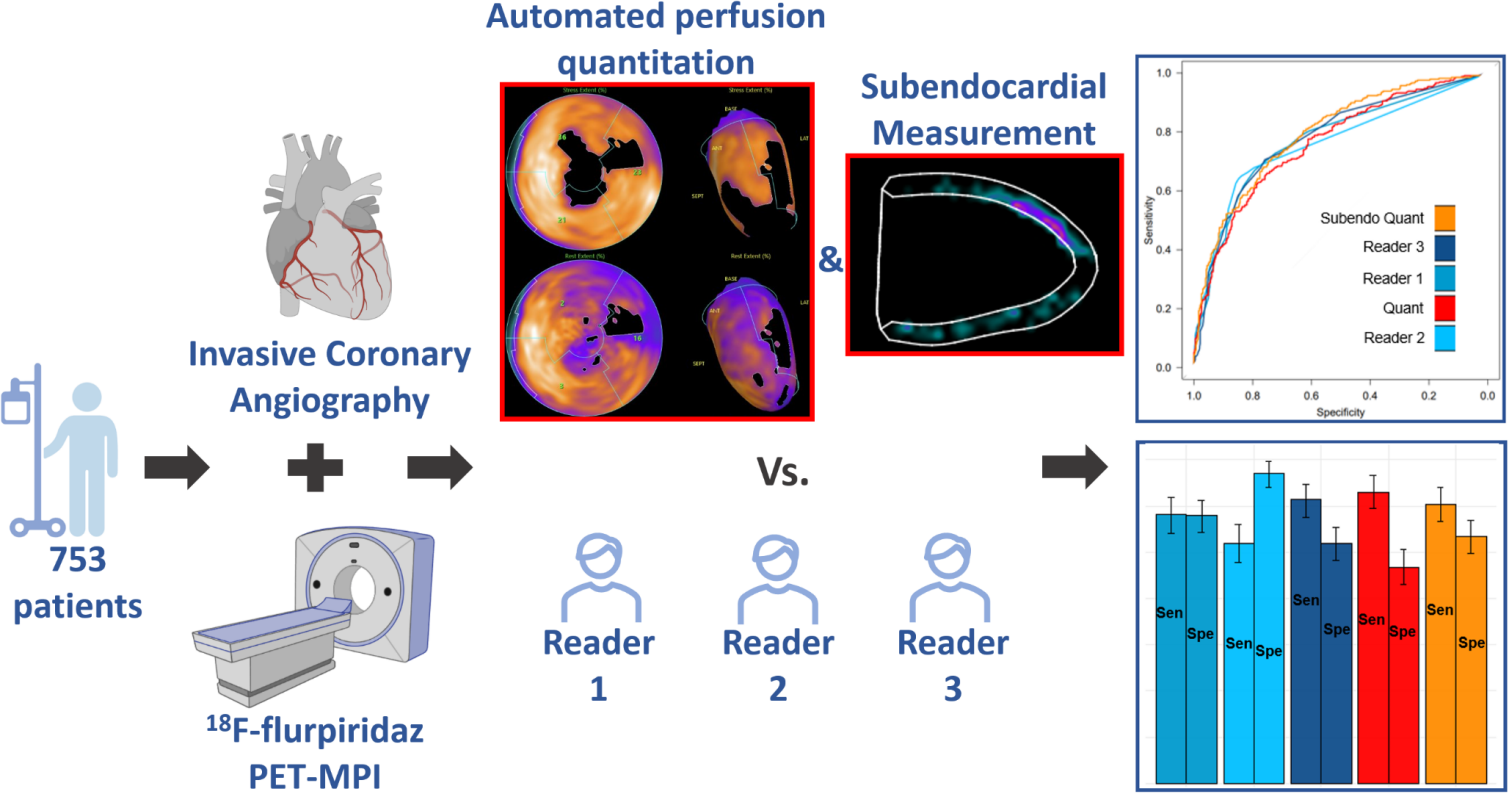
Study overview. MPI– myocardial perfusion imaging; Quant– quantitative analysis; PET– positron emission tomography; Sen– sensitivity; Spe– specificity, Subendo - subendocardial

We retrospectively analyzed data from the first of the ^18^F-flurpiridaz phase III clinical trials (Lantheus Medical Imaging, NCT01347710)(flurpiridaz-301), which enrolled 795 patients with known or suspected CAD who were referred for ICA from 2011 to 2013 at 72 sites in the United States, Canada, and Finland [1]. Patients with a history of heart transplantation, non-ischemic cardiomyopathy, history of coronary artery bypass grafting or percutaneous coronary intervention within 6 months, and unstable cardiovascular status were excluded [1]. There were 755 patient cases with evaluable PET and ICA data. One case was excluded for having only rest scan data and another for being in improper format for processing. Ultimately, 753 cases were included in this analysis. The study overview is displayed in Fig. 1. All patients enrolled in the phase III trial provided written informed consent. The study complied with the Declaration of Helsinki, and Institutional Review Board approval was obtained at each site [1].

### Flurpiridaz-301 trial design

A list of PET systems included in the flurpiridaz-301 trial is presented in Supplementary Table 1. All patients underwent a rest/stress PET imaging acquisition sequence, with the administration of ^18^F-flurpiridaz doses of 92.5–111 MBq for rest, 222–240.5 MBq for pharmacological stress, and 333–351.5 MBq for exercise stress [1, 10]. Patients undergoing exercise stress had a minimum 60-minute rest-exercise dose interval, while patients undergoing pharmacological stress were administered either regadenoson, dipyridamole, or adenosine agents before receiving their stress dose of ^18^F-flurpiridaz, with a minimum 30-minute rest-stress dose interval. Each participating site performed ICA using their respective practice protocols before sending data for quantitative measurement to a core laboratory (PERFUSE Core Laboratories and Data Coordinating Center, Boston, Massachusetts) [1].

### Angiographic abnormality criteria

The primary outcome was defined as ≥ 50% stenosis by quantitative ICA. Stenosis ≥ 50% was chosen as the primary outcome since it was the original study endpoint [1, 5]. Additionally, the quantitative coronary angiography provides lower estimates of diameter stenosis than visual assessment [11], and studies suggest that ≥ 50% stenosis is the optimal cut-off for identifying fractional flow reserve < 0.80 [12]. A secondary outcome of stenosis ≥ 70% or stenosis ≥ 50% in the left main (LM) trunk was also considered and reported.

### Visual reader analysis

In the flurpiridaz-301 trial, all PET images were interpreted by three blinded expert readers (R1, R2, R3) board-certified in nuclear cardiology [1]. Readers performed standard American Heart Association 17-segment, 5-point scoring [13], and were instructed to consider ≥ 4 for summed stress score (SSS) and ≥ 2 for summed difference score (SDS) as abnormal [14]. Qualitative diagnosis for the presence of CAD was reported on a 4-point scale: Normal, Ischemia, Ischemia plus scar (> 30% change in relative tracer uptake between stress and rest) and Scar (< 30% change in uptake between stress and rest) [1].

### Transmural and subendocardial Polar map derivation

Stress and rest myocardial contours were verified by an experienced technologist, and when necessary, were adjusted to correspond to the myocardial boundaries. All other processing was fully automated. The transmural polar maps were derived with the standard approach, utilizing the maximum count between the epicardial and endocardial borders [15]. For the subendocardial polar maps, the polar samples were defined as the maximal counts within the inner half of the myocardial wall, defined by the endocardial and epicardial boundaries. The transmural and subendocardial polar maps were computed from the same left ventricular (LV) myocardial contours.

### Automated TPD quantification

Stress and rest TPD were quantified using QPET software (Cedars-Sinai, Los Angeles, California, United States) using methods previously described [16, 17]. An enhanced research version of QPET was utilized, which enabled the creation of both transmural and subendocardial polar maps. Ischemic TPD was calculated as the difference between stress and rest TPD derived from transmural or subendocardial polar maps. Normal databases for this analysis were developed using 15 patients with visually normal studies in the final reading, from the phase II ^18^F-flurpiridaz trial [18], utilizing a previously established approach [17].

### Diagnostic performance of PET

The diagnostic performance for CAD was assessed using area under the receiver operator characteristic curve (AUC), analyzed globally and per vascular territory (left anterior descending artery [LAD], right coronary artery [RCA], and left circumflex artery [LCX]). To compare the diagnostic performance of automated and visual measures, stress TPD was compared to the SSS, and ischemic TPD was compared to the SDS reported by the three readers, in terms of their ability to detect CAD as reported by ICA. We also evaluated for differences in diagnostic performance for traditional transmural quantitation compared to subendocardial perfusion quantitation.

Since, for clinical effectiveness, the desired threshold for automatic measurements should favor sensitivity over specificity (and a higher TPD threshold results in lower sensitivity), the abnormality thresholds for TPD were determined at the highest possible TPD threshold, which still maintains greater sensitivity compared to the qualitative diagnoses based on majority rule from the readers for the presence of CAD. The quantitative thresholds were rounded to the nearest integer values as per the software’s reporting. The majority rule was established from the consensus of two out of three readers. This approach was taken to account for any biases in visual evaluation, particularly because defects are known to be more prominent in ^18^F-flurpiridaz images [19].

### Study endpoints

The primary endpoint of the study was the area under receiver operator characteristics curve (AUC) of automatically quantified subendocardial and transmural stress and ischemic TPD and its comparison to reads from expert physicians for CAD with ≥ 50% stenosis, as described above. Secondary endpoints included stenosis ≥ 70%, sensitivity and specificity of automatically quantified subendocardial and transmural stress and ischemic TPD for detection of CAD, and differences in diagnostic performance between patient subgroups of interest (male vs. female, pharmacological stress testing vs. exercise stress testing, body mass index [BMI] ≥ 30 vs. BMI < 30).

### Statistical analysis

All statistical analyses were performed with Python 3.11.5 (Python Software Foundation, Wilmington, DE, USA), Analyse-it for Microsoft Excel 5.92, build 8053.22425, and R version 4.3.2 (R Foundation for Statistical Computing, Vienna, Austria). Continuous variables that were not normally distributed are presented as medians with interquartile range (IQR). Categorical variables are presented as count and relative frequencies (percentages). Diagnostic performance was compared using Delong’s method [20]. The sensitivity and specificity of individual readers, stress TPD, and ischemic TPD were analyzed at their respective thresholds for abnormality as described above. McNemar’s test was used to compare differences in sensitivity and specificity. A two-tailed p-value of < 0.05 was considered statistically significant.

## Results

### Study population

Patient characteristics according to the presence of CAD defined as **≥** 50% or **≥** 70% stenosis are displayed in Table 1. The median age of all participants was 63 (IQR 56, 69) and 69% were male. Pharmacological stress was utilized in 71% of the total population and 55% of participants had a BMI of **≥** 30. There were 326 patients with ≥ 50% stenosis by ICA. Table 2 displays detailed results of the invasive angiograms.

**Table 1:** Patient characteristics stratified by presence of coronary artery disease (CAD)

	All participants (*n* = 753)		CAD ≥ 50% Stenosis (*n* = 326)		CAD ≥ 70% Stenosis (*n* = 210)	
Age [years]	63 [56, 69]	65 [59, 71]			65 [58, 71]	
Sex						
Male	520 (69.1%)	272 (83.4%)			179 (85.2%)	
Female	233 (30.9%)	54 (16.6%)			31 (14.8%)	
BMI [kg/m^2^]	30 [26,35]	30 [26, 34]			30 [26, 34]	
Stress Type						
Exercise	220 (29.2%)	87 (26.7%)			56 (26.7%)	
Pharmacological	533 (70.8%)	239 (73.3%)			154 (73.3%)	
Race						
White or Caucasian	617 (81.9%)	275 (84.4%)			173 (82.4%)	
Black or African American	101 (13.4%)	38 (11.7%)			29 (13.8%)	
Asian	8 (1.1%)	2 (0.6%)			1 (0.5%)	
Native Hawaiian or Other Pacific Islander	4 (0.5%)	3 (0.9%)			3 (1.4%)	
American Indian or Alaska Native	2 (0.3%)	-			-	
Other	18 (2.4%)	6 (1.8%)			3 (1.4%)	
Not Reported	3 (0.4%)	2 (0.6%)			1 (0.5%)	
Past Medical History						
Myocardial Infarction	134 (17.8%)	86 (26.4%)			52 (24.8%)	
Hypertension	618 (82.1%)	282 (86.5%)			181 (86.2%)	
Hyperlipidemia	649 (86.2%)	303 (92.9%)			192 (91.4%)	
Prior PCI	241 (32.0%)	148 (45.4%)			96 (45.7%)	
Stroke	50 (6.6%)	22 (6.7%)			17 (8.1%)	
Diabetes Mellitus	258 (34.3%)	134 (41.1%)			95 (45.2%)	
Smoking	449 (59.6%)	212 (65.0%)			137 (65.2%)	
Family History CAD	436 (57.9%)	198 (60.7%)			126 (60.0%)	

BMI– body mass index; PCI– percutaneous coronary intervention

**Table 2:** Invasive angiogram results

	Number of patients (%)
LAD stenosis	
≥ 50%	177 (23.5%)
≥ 70%	79 (10.5%)
LCX stenosis	
≥ 50%	143 (19.0%)
≥ 70%	80 (10.6%)
RCA stenosis	
≥ 50%	167 (22.2%)
≥ 70%	100 (13.3%)
LM stenosis	
≥ 50%	7 (0.9%)
≥ 70%	1 (0.1%)
1- vessel disease	
≥ 50%	196 (26.0%)
≥ 70%	163 (21.6%)
2- vessel disease	
≥ 50%	99 (13.1%)
≥ 70%	39 (5.2%)
3+- vessel disease	
≥ 50%	31 (4.1%)
≥ 70%	6 (0.8%)

LAD - left anterior descending artery; LCX - left circumflex; LM - left main; RCA - right coronary artery

### Automatic analysis

In 2.4% of stress and 4.3% of rest cases contours were manually corrected to correspond to the myocardium. All results were processed in batch mode and obtained within an average of 10 s per case. The threshold for abnormal subendocardial and transmural stress TPD was determined to be > 6%, and > 4% for ischemic TPD.

### Diagnostic performance stress TPD

**Fig. 2:**
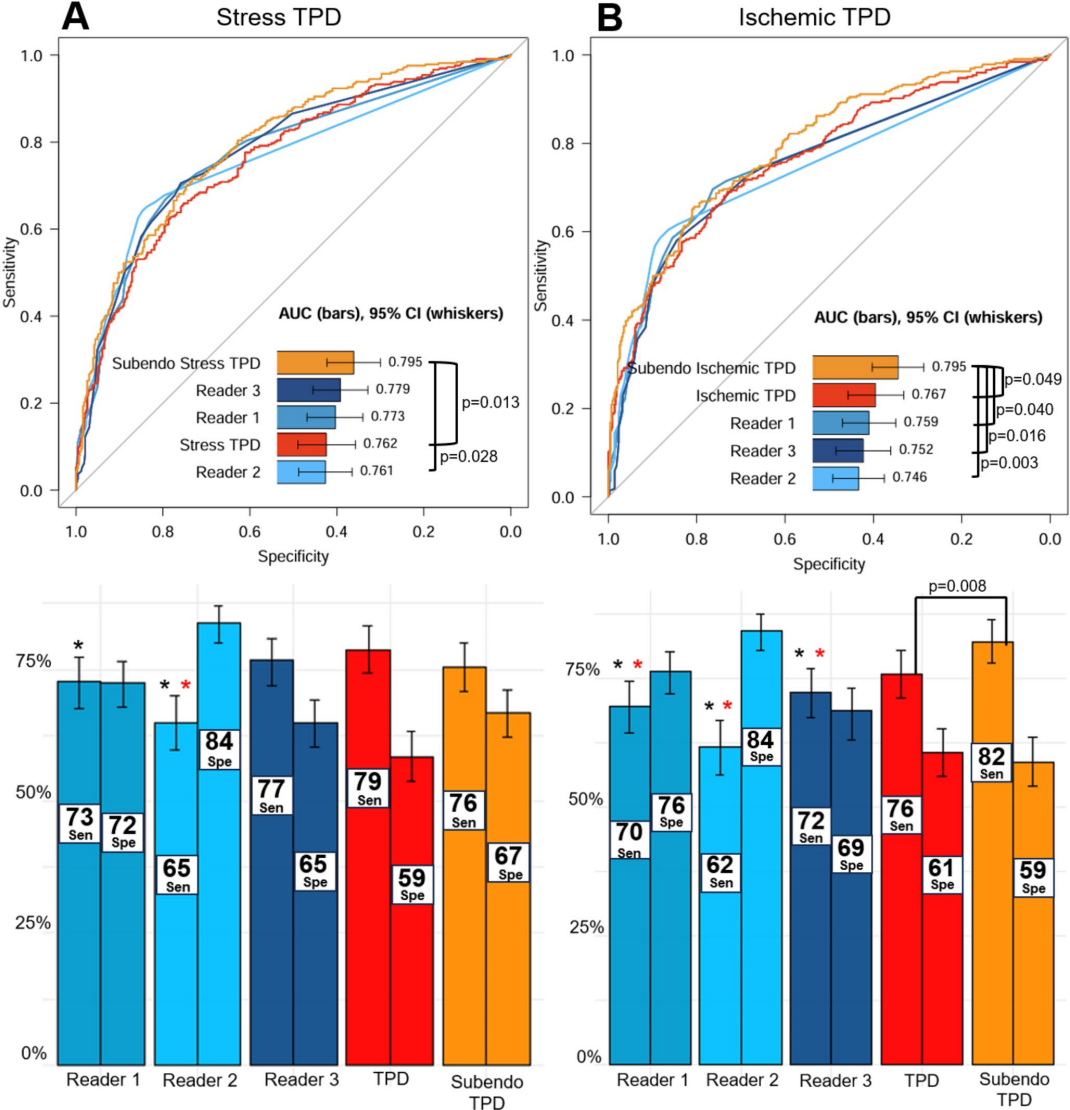
Total perfusion deficit for ≥ 50% stenosis for all participants. The diagnostic performance and Sensitivity (Sen) and Specificity (Spe) of transmural and subendocardial (subendo) stress (**A**) and ischemic (**B**) total perfusion deficit (TPD) for coronary artery disease defined as ≥ 50% stenosis is compared with expert readers’ summed stress and summed difference scores. 326 subjects were positive by invasive angiography. Only significant p-values (< 0.05) are presented in the figure. Red asterisk: *p* < 0.05 for readers versus subendocardial TPD. Black asterisk: *p* < 0.05 for readers versus transmural TPD. AUC– area under the receiver operating characteristic curve; CI– confidence interval

Figure 2A shows the diagnostic performance for CAD defined as stenosis ≥ 50%. AUC for quantified subendocardial stress TPD was higher than for transmural stress TPD and Reader 2.

Additionally, stress subendocardial TPD demonstrated higher sensitivity than 2 of the 3 the readers (Fig. 2A). Detailed results are shown in Supplementary Table 2. For the secondary endpoint, detection of ≥ 70% stenosis, AUC results for subendocardial and transmural quantitation were similar (*p* = 0.339) (Supplementary Fig. 1). Both subendocardial and transmural quantitative measures demonstrated performance that was comparable to or greater than that of the readers’ assessments for CAD (Supplementary Tables 2, 3 and 4).

The AUCs for the diagnostic performance of subendocardial and transmural stress TPD per vascular territory are shown in Supplementary Tables 5 and 6.

### Diagnostic performance ischemic TPD

Subendocardial ischemic TPD had a higher AUC than transmural ischemic TPD and all the readers (Fig. 2B). Additionally, ischemic TPD demonstrated higher sensitivity than any of the readers and transmural analysis (Fig. 2B). Detailed results are also shown in Supplementary Table 7. For the secondary endpoint, AUC results for subendocardial and transmural quantitation were similar (*p* = 0.978) (Supplementary Fig. 1). Both subendocardial and transmural quantitative measures demonstrated performance that was comparable to or greater than that of the readers’ assessments for CAD (Supplementary Tables 7, 8 and 9).

### Impact of sex on diagnostic performance

Subendocardial measurement of stress TPD showed higher AUC compared to transmural measurement in the male population (Supplementary Fig. 2). All other results were similar (Supplementary Tables 2, 3 and 4).

AUC values for subendocardial and transmural ischemic TPD were similar each other and to readers visual assessments in both sex subgroups (Supplementary Tables 7, 8 and 9).

Diagnostic performance of subendocardial and transmural stress and ischemic TPD showed no significant difference between males and females (Supplementary Table 10).

### Impact of Stress-Type on diagnostic performance

Subendocardial stress TPD measurement showed higher AUC and improved diagnostic performance compared to the SSS of R2 in the pharmacological stress population (Supplementary Fig. 3). All other results were similar (Supplementary Tables 2, 3 and 4).

Stress-type analysis showed that the AUC for subendocardial ischemic TPD was significantly higher when comparing to the SDS for R2 and R3 in the pharmacological stress patient subgroup (Supplementary Table 7). Additionally, the AUC for transmural ischemic TPD was higher than the AUC of the SDS for R2 in the pharmacological stress subgroup (Supplementary Table 9). All other comparisons of subendocardial and transmural ischemic TPD to each other and to visual assessments were not significant (Supplementary Tables 7, 8 and 9).

Diagnostic performance of subendocardial and transmural stress and ischemic TPD showed no significant difference between stress-types (Supplementary Table 10).

### Impact of BMI on diagnostic performance

Subendocardial stress TPD measurement had a significantly higher AUC than that of transmural measurement and the SSS of R2 and R3 in patients with a BMI of < 30 (Supplementary Fig. 4). All other results were similar (Supplementary Tables 2, 3 and 4).

Subendocardial ischemic TPD had a significantly higher AUC than that of transmural ischemic TPD and the SDSs of R2 and R3 in patients with a BMI of < 30 (Supplementary Table 7). It was also significantly higher than the AUC for the SDS of R2 in patients with a BMI of ≥ 30 (Supplementary Table 7). Additionally, the AUC for transmural ischemic TPD was higher than the AUC of the SDS for R2 stenosis in patients with a BMI of ≥ 30 (Supplementary Table 9). All other comparisons of subendocardial and transmural ischemic TPD to each other and to visual assessments were similar (Supplementary Tables 7, 8 and 9).

Diagnostic performance of subendocardial and transmural stress and ischemic TPD showed no significant difference between BMI categories (Supplementary Table 10).

**Fig. 3:**
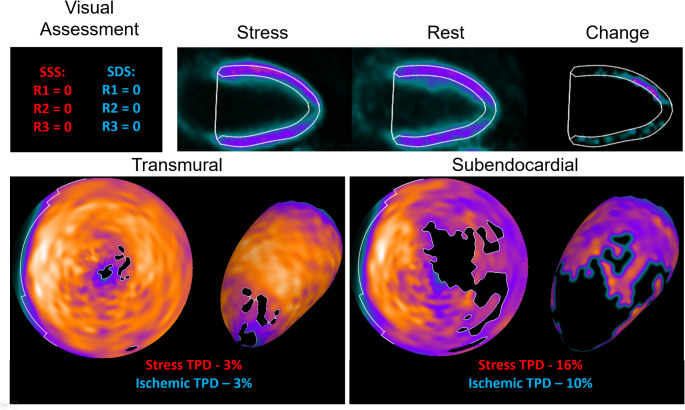
Case example. Automatic quantification of transmural and subendocardial perfusion in a 65-year-old male patient with coronary artery disease, identified by invasive angiography as having 57% stenosis in the left anterior descending artery. The top panel shows perfusion images in the vertical long axis under stress (left), at rest (middle), and the difference between stress and rest (right). Transmural perfusion quantification indicated a stress total perfusion deficit (TPD) of 3% and ischemic TPD of 3% whereas subendocardial quantification indicated a stress TPD of 16% and ischemic TPD of 10%. The three readers’ (R1, R2, R3) summed stress scores (SSS) and summed difference scores (SDS) were considered to be normal

Figure 3 and Supplementary Fig. 5 present case examples where quantitative subendocardial TPD successfully detected patients with CAD, as confirmed by ICA, while transmural TPD and visual assessments by the three readers did not.

## Discussion

We evaluated the diagnostic performance for CAD detection of automated subendocardial and transmural quantitative perfusion, for PET-MPI in the ^18^F-flurpiridaz trial. Subendocardial analysis performed better for the detection of ≥ 50% stenosis than transmural quantitative analysis or visual expert reading. Subendocardial and transmural stress and ischemic TPD achieved diagnostic performance comparable to or greater than that of the readers’ assessments in the total population as well as across different sexes, stress types, and BMI categories. Automated perfusion quantification demonstrated comparable performance to visual analysis by core expert readers in in detecting ≥ 70% stenosis. These findings demonstrated that quantifying subendocardial perfusion is feasible in the analysis of high-resolution PET tracers such as ^18^F-flurpiridaz, and provides additional information to the standard analysis, especially when evaluating moderate disease. Our approach utilizes static perfusion images, which form the basis of clinical reporting and could be easily integrated into the clinical workflow.

Subendocardial ischemia is commonly diagnosed but often not quantified routinely and is frequently not recognized by physicians [7]. However, the subendocardium is particularly susceptible to microvascular ischemia [21]. Impaired perfusion to the subendocardium has been observed in patients with hypertension, aortic stenosis, hypertrophic cardiomyopathy and chronic kidney disease [8]. Subendocardial perfusion abnormalities are also common in patients with microvascular angina who are assessed using PET-MPI [22]. A unique benefit from ^18^F-flurpiridaz is the ability to perform exercise stress; however, this precludes measurement of stress myocardial blood flow. Subendocardial perfusion analysis could potentially recover this information regarding microvascular perfusion abnormalities to help physicians target medical therapies in these populations [22]. Additionally, in hypertensive populations, lower subendocardial myocardial flow reserve has been associated with higher rates of all-cause death and heart failure hospitalization [8]. Diffuse subendocardial ischemia is a predominant mechanism for transient ischemic dilation of the left ventricle [23], a well-established marker of cardiovascular risk [24]. These findings suggest that subendocardial perfusion is a critical indicator of underlying pathophysiology and cardiovascular risk [8]. The feasibility of assessing subendocardial ischemia with PET has been reported with ^15^O-water [6, 9, 25, 26, ] ^13^N-ammonia [8, 27] and ^82^Rb [7]. In a pilot study involving sixty-six patients evaluated for CAD with ^15^O-water, neither subendocardial perfusion nor the transmural perfusion gradient improved diagnostic accuracy over transmural analysis [9], possibly due to the resolution limitation of the tracer. Diagnostic performance of subendocardial approach in static perfusion images—the basis of clinical assessment—using a high-resolution tracer such as ^18^F-flurpiridaz has not been assessed to date. Our study is the first to demonstrate that subendocardial analysis can enhance diagnostic performance compared to traditional transmural perfusion quantitation and visual assessments by expert physicians in detecting CAD in a large, multi-site population.

For the primary endpoint (≥ 50% stenosis), subendocardial perfusion quantification demonstrated a significantly higher AUC compared to transmural analysis (0.795 vs. 0.762, respectively, for stress TPD and 0.795 vs. 0.767, respectively, for ischemic TPD) reflecting its enhanced sensitivity in detecting moderate or borderline obstructive CAD. This is consistent with the known vulnerability of the subendocardium to ischemia. In contrast, for the secondary endpoint (≥ 70% stenosis), the differences in diagnostic performance between subendocardial and transmural analyses (0.819 vs. 0.808, respectively, for stress TPD and 0.810 vs. 0.810, respectively, for ischemic TPD) were not statistically significant. This may be explained by the fact that in cases of severe stenosis, perfusion deficits extend beyond the subendocardium and are more easily detectable through both transmural quantification and visual assessment. As a result, subendocardial analysis does not offer the same incremental diagnostic advantage as it does for moderate stenosis.

The key aspect of this study, in the context of the new F-18 tracer soon entering the clinical arena, is the objective quantitative analysis. Current PET-MPI interpretation relies on subjective visual assessments by physicians, which may hinder broad adoption. Substantial variability of visual assessment is still present even with highly experienced readers from the 301 trial (Fig. 2 and Supplementary Fig. 1). This variance is likely to be even more pronounced in a broad spectrum of centers without prior PET experience that will soon begin using this new tracer. Importantly, perfusion defects with ^18^F-flurpiridaz are larger due to its high extraction fraction and excellent spatial resolution, which can further increase variability. Other studies also reported the diagnostic performance for transmural myocardial blood flow measures for ^18^F-flurpiridaz [28–31]. To our knowledge, only one other study reported automated relative quantitation of ^18^F-flurpridaz to date, in a smaller cohort of patients [32]. None of these studies evaluated subendocardial perfusion.

^18^F-flurpiridaz imaging, due to short positron range and high extraction fraction, leads to larger apparent perfusion defects in patients with CAD [3, 18, 19, 33–35]. As mentioned previously, the subendocardium is affected disproportionately by the difference in perfusion patterns that results from higher extraction fraction. Consequently, clinical interpretation experience with other PET perfusion tracers may not necessarily translate directly to the interpretation of ^18^F-flurpiridaz scans. Given that the images from ^18^F-flurpiridaz differ from those produced by other PET radiotracers (which many current practitioners and clinical centers are accustomed to), the automated analysis could aid physicians in transitioning to read PET-MPI with this new tracer. This will be particularly valuable in complex cases that may initially present with ambiguous or subtle findings and those with subendocardial ischemia.

Our study has several limitations. ICA was performed to assess for coronary stenosis at each site and was utilized as an endpoint in both Phase III studies of ^18^F-flurpridaz. This may lead to a selection bias since patients with abnormal PET tend to be referred for ICA, leading to lower specificity. Furthermore, we utilized an anatomic measure for obstructive CAD rather than a functional measure (such as fractional flow reserve). However, it is important to note that ICA was interpreted using quantitative analysis which typically has greater reproducibility [36], but may have worse agreement with invasive fractional flow reserve than visual assessment [37]. Some cross-contamination from the rest scan activity is inherently present in stress scans, however the use of normal databases which contain the same effect mitigates this issue. There is potential blurring of the data due to cardiac motion, which can affect the quality of the results, especially for the subendocardial ischemia. Additionally, some myocardial contours still required adjustment by an experienced technologist; thus, the quantification process is not yet entirely automated. The data from the phase III ^18^F-flurpiridaz clinical trial did not include paired test/retest measurements, so we were not able to assess test-retest variability of the new technique.

## Conclusion

Subendocardial analysis of ischemic perfusion improved the detection of CAD compared to transmural quantitative analysis or visual expert reading. Transmural perfusion quantification demonstrated comparable performance to visual analysis by core expert readers in the multi-site clinical trial. The subendocardial and transmural quantitative measures can be derived automatically with minimal user interaction. The incorporation of TPD quantitative measures using ^18^F-flurpiridaz could standardize and potentially enhance the diagnosis of CAD, aiding in the widespread clinical adoption of this novel tracer.

## Electronic supplementary material

Below is the link to the electronic supplementary material.

Supplementary Material 1

Supplementary Material 1

## Data Availability

To the extent allowed by data sharing agreements and IRB protocols, the data from this manuscript will be shared upon written request.

## Abbreviations

CAD: Coronary artery disease
PET: Positron emission tomography
MPI: Myocardial perfusion imaging
TPD: Total perfusion deficit
ICA: Invasive coronary angiography
AUC: Area under the receiver operating characteristic curve
SSS: Summed stress score
SDS: Summed difference score
