# Supplementary material for: Subendocardial quantification enhances coronary artery disease detection in 18F-flurpiridaz PET
Source: Eur J Nucl Med Mol Imaging. 2025 Mar 5;52(9):3342–52. doi: 10.1007/s00259-025-07174-6 (PMC12221820; doi:10.1007/s00259-025-07174-6)
Supplement: Supplementary file 2 — Supplementary Material 1 [file 259_2025_7174_MOESM2_ESM.docx]

**Subendocardial Quantification Enhances Coronary Artery Disease Detection in ^18^F-flurpiridaz PET**

Valerie Builoff, BS^a^, Mark Lemley, BS^a^, Robert JH Miller, MD^a,b^, Hidesato Fujito, MD, PhD^a,c^, Giselle Ramirez, BS^a^, Paul Kavanagh, MS^a^, Christopher Buckley, PhD^d^, Marcelo Di Carli, MD^e^, Daniel S. Berman, MD^a^, Piotr Slomka, PhD^a^

1. Departments of Medicine (Division of Artificial Intelligence in Medicine), and Imaging, Cedars-Sinai Medical Center, Los Angeles, CA
2. Department of Cardiac Sciences, University of Calgary, Calgary AB, Canada
3. Department of Cardiology, Nihon University Itabashi Hospital, Tokyo, Japan
4. GE Healthcare, Pharmaceutical Diagnostics, Buckinghamshire, UK
5. Division of Nuclear Medicine and Molecular Imaging, Department of Radiology, and Division of Cardiovascular Medicine, Department of Medicine, Brigham and Women's Hospital, Boston, MA, USA

**Address for Correspondence:**

Piotr Slomka, PhD

Cedars-Sinai Medical Center

6500 Wilshire Boulevard

Los Angeles, California 90048

Phone: 310-423-4348 Fax: 310-423-0173

Email: [Piotr.Slomka@cshs.org](mailto:Piotr.Slomka@cshs.org)

**Supplementary Table 1. PET devices in ^18^F-flurpiridaz phase III clinical trial**

| **PET System** | **Number of patients (%)** |
| --- | --- |
| GE Advance | 1 (0.1%) |
| GE Discovery 600 | 24 (3.2%) |
| GE Discovery 690 | 26 (3.5%) |
| GE Discovery RX | 11 (1.5%) |
| GE Discovery ST | 89 (11.8%) |
| GE Discovery STE | 49 (6.5%) |
| Philips Gemini | 70 (9.3%) |
| Siemens Biograph | 252 (33.5%) |
| Siemens Biograph mCT | 15 (2.0%) |
| Siemens ECAT | 3 (0.4%) |
| Siemens ECAT Accel | 190 (25.2%) |
| Siemens ECAT Exact | 1 (0.1%) |
| Siemens ECAT HR+ | 22 (2.9%) |

**Supplementary Table 2. Stress total perfusion deficit (TPD) for ≥50% stenosis.**

|  | **CAD ≥50% Stenosis**  **(n=326)** | | | | | | | | | |
| --- | --- | --- | --- | --- | --- | --- | --- | --- | --- | --- |
|  | Reader 1 | | Reader 2 | | Reader 3 | | Stress TPD | | Subendocardial Stress TPD | |
|  | AUC  [95% CI] | p-value | AUC  [95% CI] | p-value | AUC  [95% CI] | p-value | AUC  [95% CI] | p-value | AUC  [95% CI] | p-value |
| All Subjects |  |  |  |  |  |  |  |  |  |  |
|  | 0.773  [0.739-0.806] | 0.154 | 0.761  [0.729-0.793] | **0.028** | 0.779  [0.746-0.812] | 0.351 | 0.762  [0.728-0.797] | **0.013** | 0.795  [0.763-0.827] | **-** |
| Sex |  |  |  |  |  |  |  |  |  |  |
| Male | 0.755  [0.715-0.796] | 0.212 | 0.758  [0.720-0.796] | 0.244 | 0.761  [0.720-0.801] | 0.364 | 0.738  [0.696-0.781] | **0.013** | 0.778  [0.739-0.818] | - |
| Female | 0.768  [0.694-0.843] | 0.644 | 0.739  [0.667-0.812] | 0.762 | 0.788  [0.716-0.861] | 0.394 | 0.753  [0.679-0.826] | 0.962 | 0.751  [0.674-0.828] | - |
| Stress Type |  |  |  |  |  |  |  |  |  |  |
| Exercise | 0.727  [0.658-0.796] | 0.190 | 0.746  [0.684-0.808] | 0.481 | 0.753  [0.687-0.819] | 0.674 | 0.723  [0.653-0.792] | 0.069 | 0.767  [0.701-0.833] | - |
| Pharma | 0.790  [0.752-0.828] | 0.322 | 0.766  [0.728-0.804] | **0.017** | 0.789  [0.750-0.828] | 0.327 | 0.778  [0.739-0.818] | 0.054 | 0.808  [0.772-0.844] | **-** |
| BMI |  |  |  |  |  |  |  |  |  |  |
| ≥30 | 0.785  [0.741-0.830] | 0.873 | 0.774  [0.731-0.817] | 0.494 | 0.807  [0.763-0.850] | 0.447 | 0.776  [0.731-0.822] | 0.492 | 0.789  [0.745-0.833] | **-** |
| <30 | 0.758  [0.708-0.808] | 0.061 | 0.742  [0.693-0.791] | **0.010** | 0.747  [0.695-0.799] | **0.027** | 0.753  [0.702-0.805] | **0.011** | 0.800  [0.753-0.847] | **-** |

Area under the receiver operating curve (AUC) values for the diagnostic performance of transmural and subendocardial stress total perfusion deficit and the summed stress scores of three readers for coronary artery disease (CAD) defined as ≥50% stenosis. P-values are displayed as comparisons to subendocardial stress TPD. Significant p-values are shown in bold.

BMI – body mass index; CI – confidence interval; Pharma – pharmacological

**Supplementary Table 3. Stress total perfusion deficit (TPD) for ≥70% stenosis.**

|  | **CAD ≥70% Stenosis**  **(n=210)** | | | | | | | | | |
| --- | --- | --- | --- | --- | --- | --- | --- | --- | --- | --- |
|  | Reader 1 | | Reader 2 | | Reader 3 | | Stress TPD | | Subendocardial Stress TPD | |
|  | AUC  [95% CI] | p-value | AUC  [95% CI] | p-value | AUC  [95% CI] | p-value | AUC  [95% CI] | p-value | AUC  [95% CI] | p-value |
| All Subjects |  |  |  |  |  |  |  |  |  |  |
|  | 0.813  [0.779-0.847] | 0.669 | 0.799  [0.764-0.834] | 0.174 | 0.826  [0.793-0.859] | 0.649 | 0.808  [0.774-0.843] | 0.339 | 0.819  [0.786-0.853] | **-** |
| Sex |  |  |  |  |  |  |  |  |  |  |
| Male | 0.784  [0.744-0.825] | 0.615 | 0.786  [0.745-0.826] | 0.655 | 0.812  [0.774-0.850] | 0.285 | 0.784  [0.743-0.825] | 0.530 | 0.793  [0.752-0.834] | - |
| Female | 0.854  [0.775-0.934] | 0.553 | 0.815  [0.732-0.899] | 0.727 | 0.827  [0.738-0.916] | 0.949 | 0.814  [0.730-0.899] | 0.613 | 0.830  [0.752-0.908] | - |
| Stress Type |  |  |  |  |  |  |  |  |  |  |
| Exercise | 0.801  [0.733-0.869] | 0.175 | 0.812  [0.747-0.877] | 0.320 | 0.831  [0.770-0.893] | 0.722 | 0.812  [0.750-0.873] | 0.197 | 0.843  [0.784-0.902] | - |
| Pharma | 0.818  [0.779-0.857] | 0.740 | 0.794  [0.752-0.836] | 0.255 | 0.824  [0.785-0.863] | 0.520 | 0.807  [0.766-0.848] | 0.646 | 0.813  [0.773-0.852] | **-** |
| BMI |  |  |  |  |  |  |  |  |  |  |
| ≥30 | 0.815  [0.768-0.862] | 0.857 | 0.800  [0.751-0.848] | 0.586 | 0.822  [0.776-0.867] | 0.632 | 0.808  [0.760-0.856] | 0.840 | 0.811  [0.765-0.858] | **-** |
| <30 | 0.806  [0.757-0.856] | 0.308 | 0.796  [0.745-0.847] | 0.116 | 0.832  [0.783-0.880] | 0.861 | 0.809  [0.760-0.859] | 0.243 | 0.828  [0.780-0.876] | **-** |

Area under the receiver operating curve (AUC) values for the diagnostic performance of transmural and subendocardial stress total perfusion deficit and the summed stress scores of three readers for coronary artery disease (CAD) defined as ≥70% stenosis. P-values are displayed as comparisons to subendocardial stress TPD.

BMI – body mass index; CI – confidence interval; Pharma - pharmacological

**Supplementary Table 4. Comparison to transmural stress total perfusion deficit (TPD).**

|  | **Comparison to Transmural Stress TPD** | | | | | | | |
| --- | --- | --- | --- | --- | --- | --- | --- | --- |
|  | Reader 1 | | Reader 2 | | Reader 3 | | Subendocardial Stress TPD | |
|  | ≥50% | ≥70% | ≥50% | ≥70% | ≥50% | ≥70% | ≥50% | ≥70% |
| All Subjects |  |  |  |  |  |  |  |  |
|  | 0.427 | 0.703 | 0.918 | 0.514 | 0.261 | 0.194 | **0.013** | 0.339 |
| Sex |  |  |  |  |  |  |  |  |
| Male | 0.282 | 0.992 | 0.248 | 0.926 | 0.193 | 0.081 | **0.013** | 0.530 |
| Female | 0.609 | 0.268 | 0.709 | 0.980 | 0.319 | 0.742 | 0.962 | 0.613 |
| Stress Type |  |  |  |  |  |  |  |  |
| Exercise | 0.875 | 0.648 | 0.388 | 0.980 | 0.317 | 0.471 | 0.069 | 0.197 |
| Pharma | 0.426 | 0.434 | 0.463 | 0.436 | 0.529 | 0.289 | 0.054 | 0.646 |
| BMI |  |  |  |  |  |  |  |  |
| ≥30 | 0.624 | 0.692 | 0.907 | 0.680 | 0.151 | 0.489 | 0.492 | 0.840 |
| <30 | 0.801 | 0.862 | 0.579 | 0.456 | 0.768 | 0.253 | **0.011** | 0.243 |

Comparison of area under the receiver operating characteristic curve (AUC) to transmural stress total perfusion deficit AUC. P-values are displayed for coronary artery disease defined as ≥50% and ≥70% stenosis. Significant p-values are shown in bold.

BMI – body mass index; Pharma – pharmacological

**Supplementary Table 5. Per-vessel diagnostic performance for** ≥**50% stenosis.**

|  | **CAD ≥50% Stenosis**  **(n=326)** | | |
| --- | --- | --- | --- |
|  | Stress TPD  AUC  [95% CI] | Subendocardial Stress TPD  AUC  [95% CI] | p-value |
| Territory |  |  |  |
| RCA  (n=167) | 0.799  [0.758–0.841] | 0.815  [0.778–0.852] | 0.333 |
| LAD  (n=143) | 0.707  [0.661–0.754] | 0.751  [0.706–0.796] | **0.007** |
| LCX  (n=177) | 0.666  [0.611–0.721] | 0.679  [0.626–0.732] | 0.502 |

Per-vessel diagnostic performance of transmural and subendocardial stress total perfusion deficit (TPD) for coronary artery disease (CAD) defined as ≥50% stenosis. Significant p-values are shown in bold.

AUC – area under the receiver operating curve; CI – confidence interval; LAD - left anterior descending artery; LCX - left circumflex;  RCA - right coronary artery

**Supplementary Table 6. Per-vessel diagnostic performance for** ≥**70% stenosis.**

|  | **CAD ≥70% Stenosis**  **(n=210)** | | |
| --- | --- | --- | --- |
|  | Stress TPD  AUC  [95% CI] | Subendocardial Stress TPD  AUC  [95% CI] | p-value |
| Territory |  |  |  |
| RCA  (n=100) | 0.883  [0.847–0.919] | 0.862  [0.823–0.901] | 0.151 |
| LAD  (n=83) | 0.816  [0.759–0.872] | 0.831  [0.774–0.888] | 0.353 |
| LCX  (n=83) | 0.772  [0.714–0.830] | 0.749  [0.687–0.810] | 0.344 |

Per-vessel diagnostic performance of transmural and subendocardial stress total perfusion deficit (TPD) for coronary artery disease (CAD) defined as ≥70% stenosis.

AUC – area under the receiver operating curve; CI – confidence interval; LAD - left anterior descending artery; LCX - left circumflex; RCA - right coronary artery

**Supplementary Table 7. Ischemic total perfusion deficit (TPD) for ≥50% stenosis.**

|  | **CAD ≥50% Stenosis**  **(n=326)** | | | | | | | | | |
| --- | --- | --- | --- | --- | --- | --- | --- | --- | --- | --- |
|  | Reader 1 | | Reader 2 | | Reader 3 | | Ischemic TPD | | Subendocardial Ischemic TPD | |
|  | AUC  [95% CI] | p-value | AUC  [95% CI] | p-value | AUC  [95% CI] | p-value | AUC  [95% CI] | p-value | AUC  [95% CI] | p-value |
| All Subjects |  |  |  |  |  |  |  |  |  |  |
|  | 0.759  [0.726-0.792] | **0.040** | 0.746  [0.714-0.778] | **0.003** | 0.752  [0.718-0.786] | **0.016** | 0.767  [0.733-0.802] | **0.049** | 0.795  [0.763-0.827] | - |
| Sex |  |  |  |  |  |  |  |  |  |  |
| Male | 0.753  [0.714-0.792] | 0.188 | 0.751  [0.714-0.788] | 0.135 | 0.746  [0.705-0.786] | 0.102 | 0.755  [0.714-0.796] | 0.143 | 0.780  [0.741-0.819] | - |
| Female | 0.744  [0.669-0.819] | 0.695 | 0.709  [0.635-0.782] | 0.204 | 0.744  [0.666-0.822] | 0.726 | 0.749  [0.677-0.822] | 0.735 | 0.760  [0.684-0.836] | - |
| Stress Type |  |  |  |  |  |  |  |  |  |  |
| Exercise | 0.721  [0.654-0.788] | 0.210 | 0.723  [0.661-0.785] | 0.218 | 0.731  [0.665-0.797] | 0.387 | 0.717  [0.646-0.788] | 0.098 | 0.765  [0.698-0.832] | - |
| Pharma | 0.775  [0.737-0.813] | 0.079 | 0.754  [0.717-0.791] | **0.003** | 0.761  [0.720-0.801] | **0.011** | 0.792  [0.753-0.830] | 0.244 | 0.810  [0.774-0.846] | - |
| BMI |  |  |  |  |  |  |  |  |  |  |
| ≥30 | 0.768  [0.723-0.812] | 0.188 | 0.749  [0.706-0.792] | **0.031** | 0.771  [0.726-0.817] | 0.252 | 0.796  [0.752-0.840] | 0.877 | 0.799  [0.756-0.843] | - |
| <30 | 0.748  [0.698-0.798] | 0.098 | 0.739  [0.691-0.787] | **0.037** | 0.730  [0.677-0.782] | **0.020** | 0.740  [0.688-0.793] | **0.017** | 0.791  [0.743-0.839] | - |

Area under the receiver operating curve (AUC) values for the diagnostic performance of transmural and subendocardial ischemic total perfusion deficit (TPD) and the summed difference scores of three readers for coronary artery disease (CAD) defined as ≥50% stenosis. P-values are displayed as comparisons to subendocardial ischemic TPD. Significant p-values are shown in bold.

BMI – body mass index; CI – confidence interval; Pharma - pharmacological

**Supplementary Table 8. Ischemic total perfusion deficit (TPD) for ≥70% stenosis.**

|  | **CAD ≥70% Stenosis**  **(n=210)** | | | | | | | | | |
| --- | --- | --- | --- | --- | --- | --- | --- | --- | --- | --- |
|  | Reader 1 | | Reader 2 | | Reader 3 | | Ischemic TPD | | Subendocardial Ischemic TPD | |
|  | AUC  [95% CI] | p-value | AUC  [95% CI] | p-value | AUC  [95% CI] | p-value | AUC  [95% CI] | p-value | AUC  [95% CI] | p-value |
| All Subjects |  |  |  |  |  |  |  |  |  |  |
|  | 0.789  [0.752-0.825] | 0.247 | 0.780  [0.743-0.816] | 0.083 | 0.797  [0.761-0.834] | 0.495 | 0.810  [0.775-0.845] | 0.978 | 0.810  [0.775-0.845] | **-** |
| Sex |  |  |  |  |  |  |  |  |  |  |
| Male | 0.766  [0.723-0.808] | 0.528 | 0.769  [0.727-0.810] | 0.614 | 0.786  [0.745-0.828] | 0.735 | 0.792  [0.750-0.833] | 0.436 | 0.779  [0.736-0.822] | - |
| Female | 0.832  [0.745-0.918] | 0.687 | 0.790  [0.702-0.879] | 0.139 | 0.799  [0.702-0.896] | 0.255 | 0.816  [0.733-0.899] | 0.256 | 0.849  [0.778-0.919] | - |
| Stress Type |  |  |  |  |  |  |  |  |  |  |
| Exercise | 0.792  [0.722-0.862] | 0.356 | 0.785  [0.717-0.853] | 0.270 | 0.795  [0.727-0.864] | 0.447 | 0.792  [0.722-0.862] | 0.269 | 0.827  [0.761-0.894] | - |
| Pharma | 0.789  [0.746-0.832] | 0.393 | 0.777  [0.734-0.820] | 0.124 | 0.797  [0.754-0.841] | 0.654 | 0.818  [0.777-0.858] | 0.451 | 0.806  [0.765-0.847] | **-** |
| BMI |  |  |  |  |  |  |  |  |  |  |
| ≥30 | 0.786  [0.735-0.837] | 0.415 | 0.773  [0.723-0.823] | 0.167 | 0.787  [0.737-0.836] | 0.428 | 0.819  [0.772-0.865] | 0.517 | 0.807  [0.759-0.855] | **-** |
| <30 | 0.789  [0.736-0.841] | 0.355 | 0.784  [0.732-0.837] | 0.268 | 0.810  [0.757-0.864] | 0.937 | 0.801  [0.748-0.854] | 0.570 | 0.813  [0.761-0.864] | **-** |

Area under the receiver operating curve (AUC) values for the diagnostic performance of transmural and subendocardial ischemic total perfusion deficit and the summed difference scores of three readers for coronary artery disease (CAD) defined as ≥70% stenosis. P-values are displayed as comparisons to subendocardial ischemic TPD.

BMI – body mass index; CI – confidence interval; Pharma – pharmacological

**Supplementary Table 9. Comparison of area under the receiver operating characteristic curve to transmural ischemic TPD.**

|  | **Comparison to Transmural Ischemic TPD** | | | | | | | |
| --- | --- | --- | --- | --- | --- | --- | --- | --- |
|  | Reader 1 | | Reader 2 | | Reader 3 | | Subendocardial Ischemic TPD | |
|  | ≥50% | ≥70% | ≥50% | ≥70% | ≥50% | ≥70% | ≥50% | ≥70% |
| All Subjects |  |  |  |  |  |  |  |  |
|  | 0.592 | 0.175 | 0.170 | 0.057 | 0.359 | 0.445 | **0.049** | 0.978 |
| Sex |  |  |  |  |  |  |  |  |
| Male | 0.907 | 0.140 | 0.812 | 0.200 | 0.630 | 0.765 | 0.143 | 0.436 |
| Female | 0.882 | 0.087 | 0.269 | 0.140 | 0.881 | 0.619 | 0.735 | 0.256 |
| Stress Type |  |  |  |  |  |  |  |  |
| Exercise | 0.898 | 0.993 | 0.848 | 0.842 | 0.692 | 0.929 | 0.098 | 0.269 |
| Pharma | 0.331 | 0.099 | **0.032** | **0.021** | 0.083 | 0.262 | 0.244 | 0.451 |
| BMI |  |  |  |  |  |  |  |  |
| ≥30 | 0.163 | 0.118 | **0.031** | **0.035** | 0.262 | 0.160 | 0.877 | 0.517 |
| <30 | 0.729 | 0.590 | 0.962 | 0.474 | 0.651 | 0.697 | **0.017** | 0.570 |

P-values are displayed for coronary artery disease defined as ≥50% and ≥70% stenosis. Significant p-values are shown in bold.

BMI – body mass index; Pharma – pharmacological

**Supplementary Table 10. Comparison of area under the receiver operating characteristic curve between subgroups.**

|  | Transmural Stress TPD | | Subendocardial Stress TPD | | Transmural Ischemic TPD | | Subendocardial Ischemic TPD | |
| --- | --- | --- | --- | --- | --- | --- | --- | --- |
|  | ≥50% | ≥70% | ≥50% | ≥70% | ≥50% | ≥70% | ≥50% | ≥70% |
| Male  vs.  Female | 0.742 | 0.529 | 0.539 | 0.412 | 0.893 | 0.607 | 0.640 | 0.098 |
| Exercise  vs.  Pharma | 0.171 | 0.891 | 0.287 | 0.400 | 0.070 | 0.531 | 0.245 | 0.596 |
| ≥30 BMI  vs.  <30 BMI | 0.509 | 0.968 | 0.738 | 0.631 | 0.110 | 0.621 | 0.800 | 0.870 |

P-values are displayed for coronary artery disease defined as ≥50% and ≥70% stenosis.

BMI – body mass index; Pharma – pharmacological

**Supplementary Figure 1. Total perfusion deficit for ≥70% stenosis for all participants.**

**
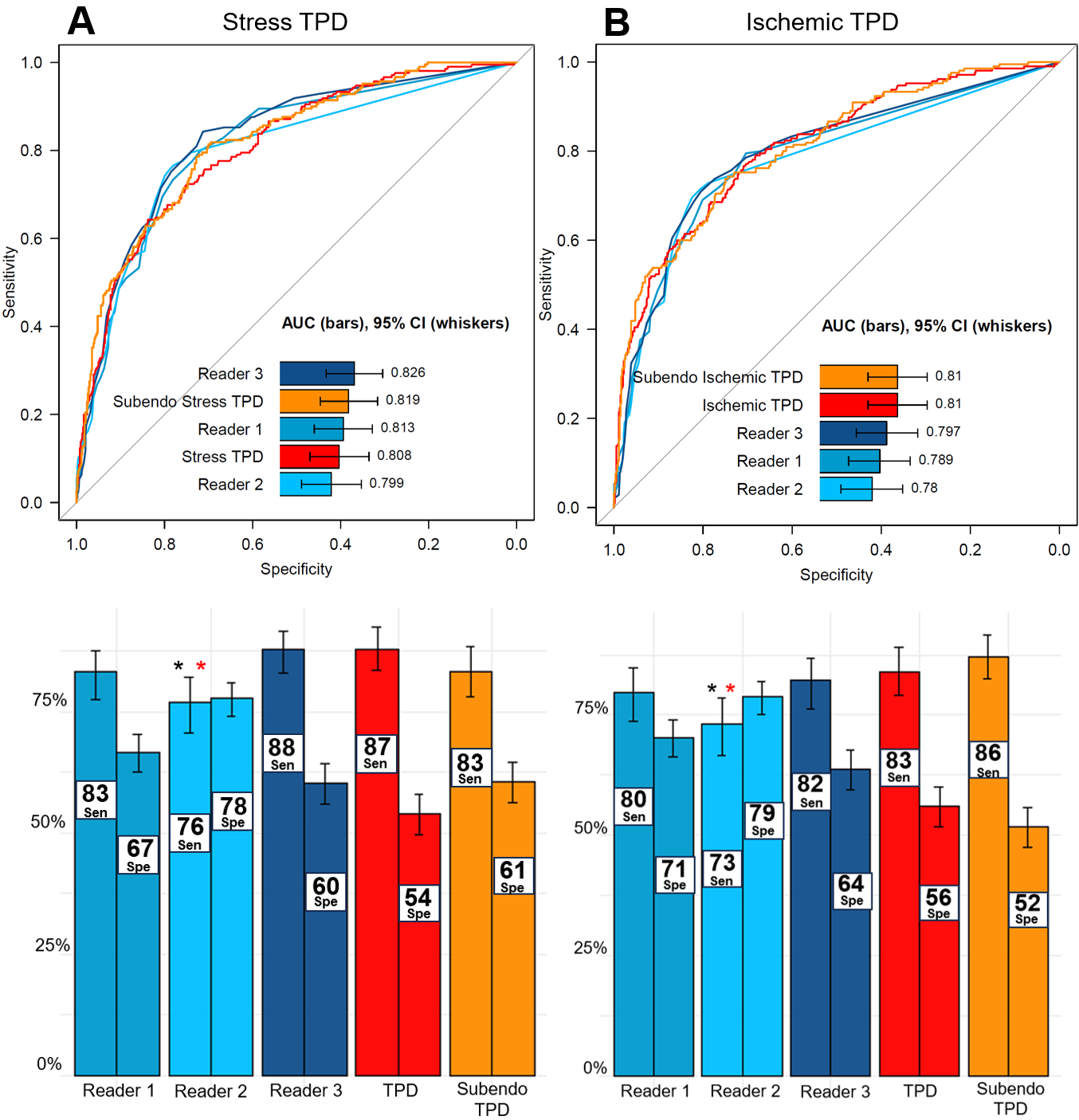
**

**Supplementary Figure 1. Total perfusion deficit for ≥70% stenosis for all participants.** The diagnostic performance and Sensitivity (Sen) and Specificity (Spe) of transmural and subendocardial (subendo) stress (A) and ischemic (B) total perfusion deficit (TPD) for coronary artery disease defined as ≥70% stenosis is compared with expert readers’ summed stress and summed difference scores. 210 subjects were positive by invasive angiography. Only significant p-values (<0.05) are presented in the figure. Red asterisk: p<0.05 for readers versus subendocardial TPD. Black asterisk: p<0.05 for readers versus transmural TPD. AUC – area under the receiver operating curve; CI – confidence interval.

**Supplementary Figure 2. Transmural and subendocardial stress total perfusion deficit (TPD) per gender.**

**
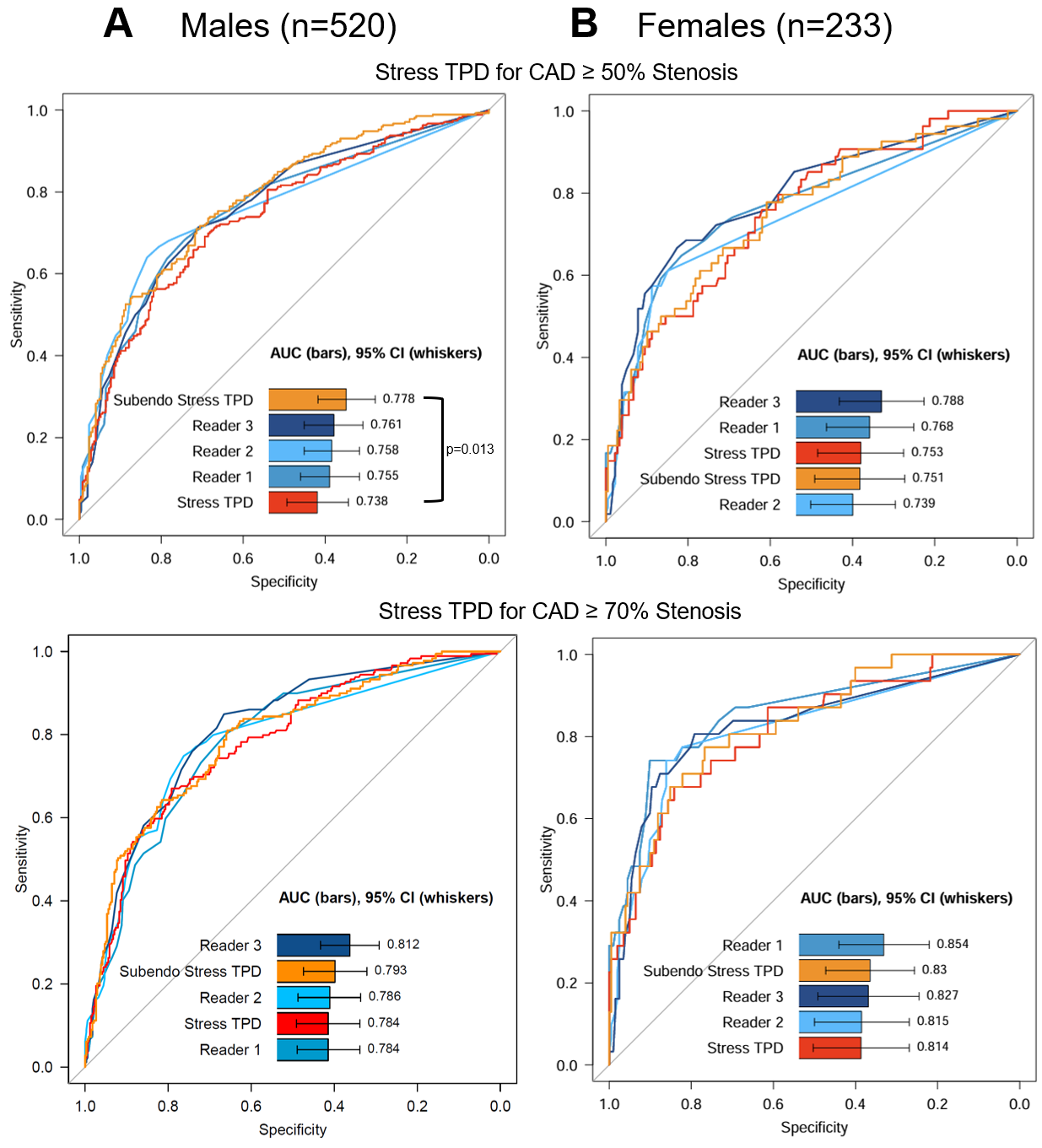
**

**Supplementary Figure 2. Transmural and subendocardial stress total perfusion deficit (TPD) per gender.** The diagnostic performance of transmural and subendocardial (subendo) stress total perfusion deficit for Males (A) and Females (B) is compared with expert readers’ summed stress scores. For the male population, 272 were positive for CAD ≥50% stenosis and 179 were positive for CAD ≥70% stenosis by invasive angiography. For the female population, 54 were positive for CAD ≥50% stenosis and 31 were positive for CAD ≥70% stenosis by invasive angiography. Only significant p-values (<0.05) are presented in the figure. AUC – area under the receiver operating curve; CI – confidence interval.

**Supplementary Figure 3. Transmural and subendocardial stress total perfusion deficit (TPD) per stress-type.**

**
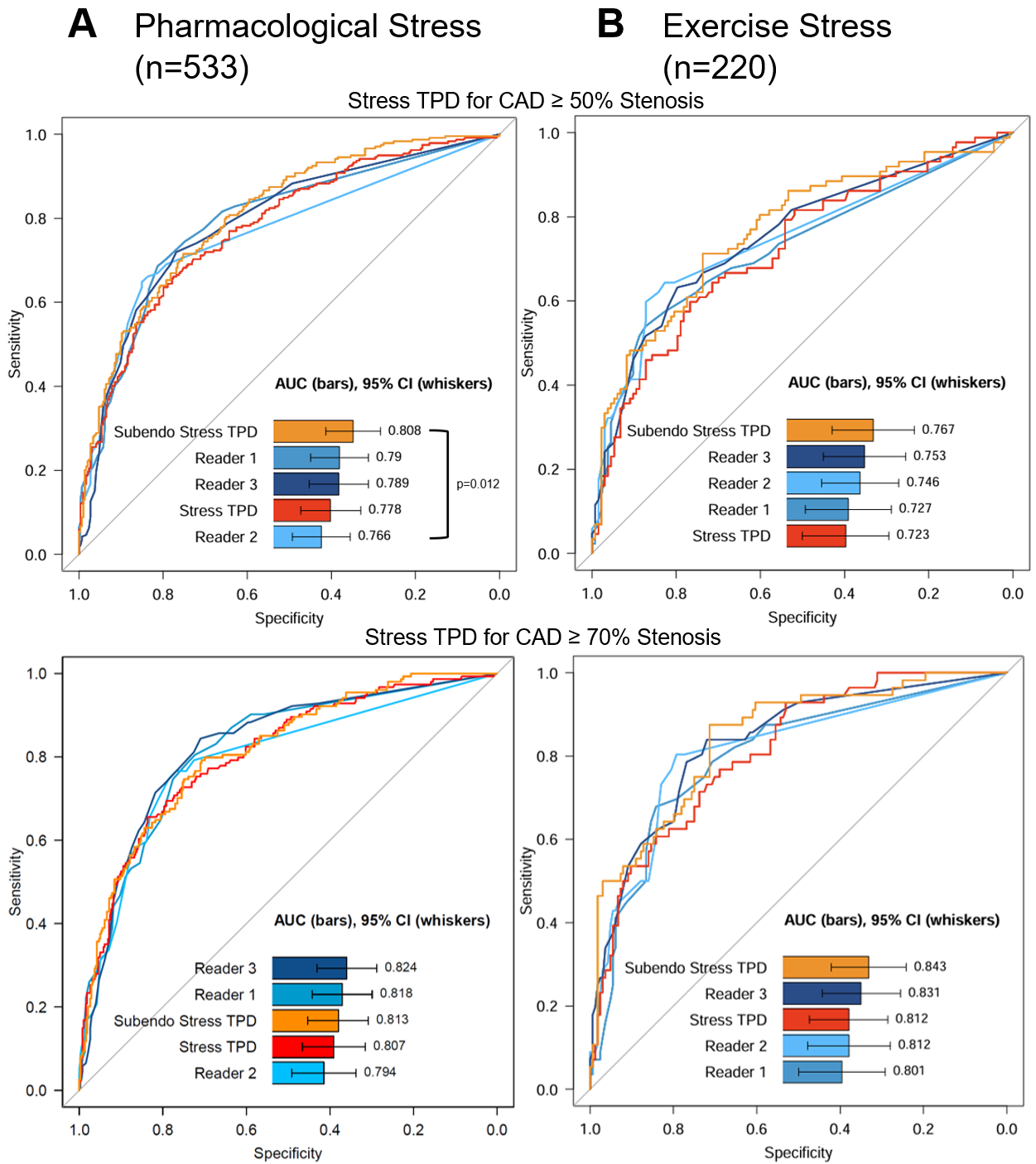
**

**Supplementary Figure 3. Transmural and subendocardial stress total perfusion deficit (TPD) per stress-type.** The diagnostic performance of transmural and subendocardial (subendo) stress TPD for patients undergoing Pharmacological Stress (A) and Exercise Stress (B) is compared with expert readers’ summed stress scores. Out of participants who underwent pharmacological stress, 239 were positive for CAD ≥50% stenosis and 154 were positive for CAD ≥70% stenosis by invasive angiography. Out of participants who underwent exercise stress, 87 were positive for CAD ≥50% stenosis and 56 were positive for CAD ≥70% stenosis by invasive angiography. Only significant p-values (<0.05) are presented in the figure. AUC – area under the receiver operating curve; CI – confidence interval.

**Supplementary Figure 4. Transmural and subendocardial stress total perfusion deficit (TPD) per BMI.**

**
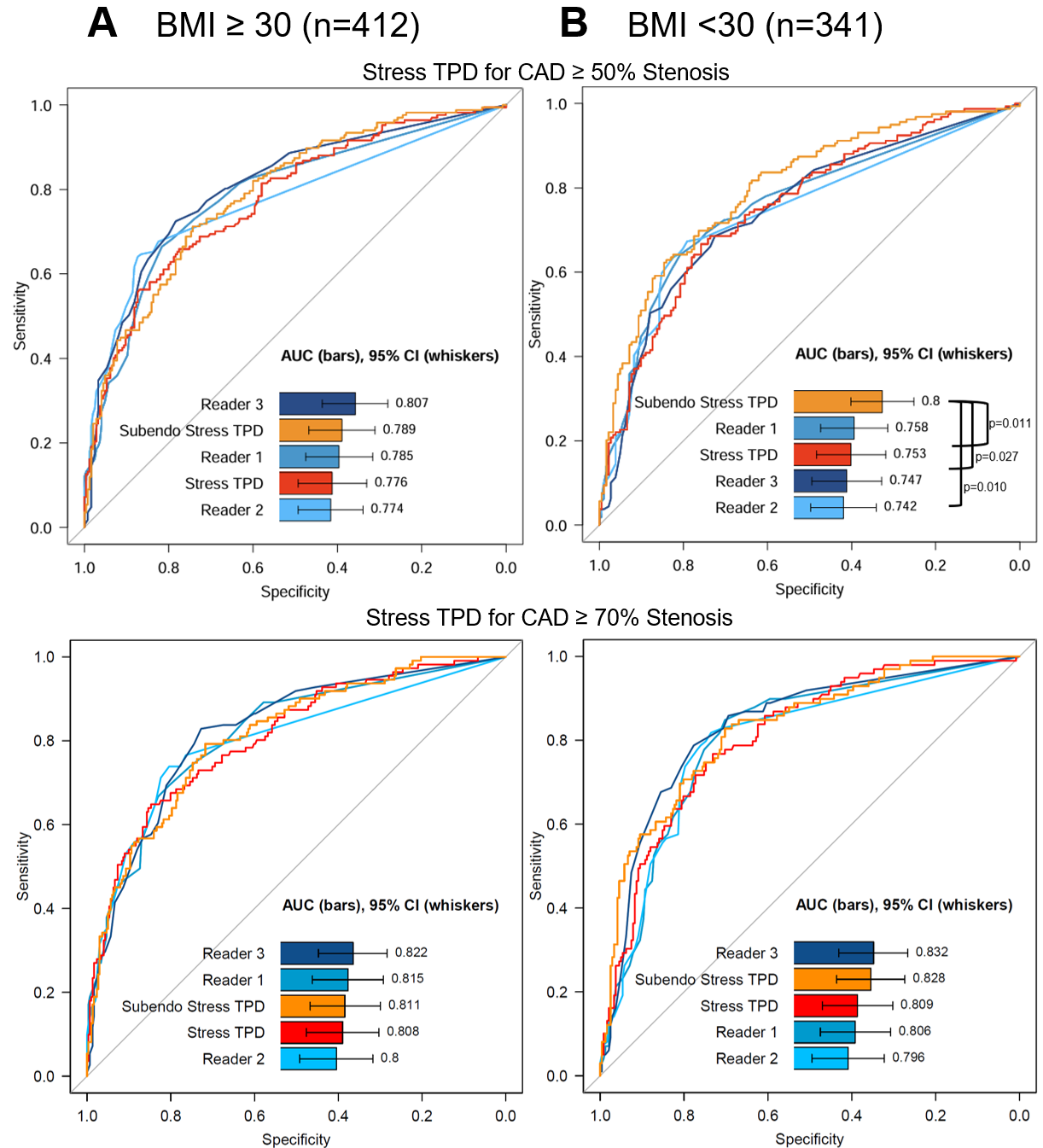
**

**Supplementary Figure 4. Transmural and subendocardial stress total perfusion deficit (TPD) per BMI.** The diagnostic performance of transmural and subendocardial (subendo) stress TPD for patients with a body mass index (BMI) ≥30 (A) and <30 (B) is compared with expert readers’ summed stress scores. Out of participants with a BMI ≥30, 167 were positive for CAD ≥50% stenosis and 111 were positive for CAD ≥70% stenosis by invasive angiography. Out of participants with a BMI <30, 159 were positive for CAD ≥50% stenosis and 99 were positive for CAD ≥70% stenosis by invasive angiography. Only significant p-values (<0.05) are presented in the figure. AUC – area under the receiver operating curve; CI – confidence interval.

**Supplementary Figure 5. Case example**.

**
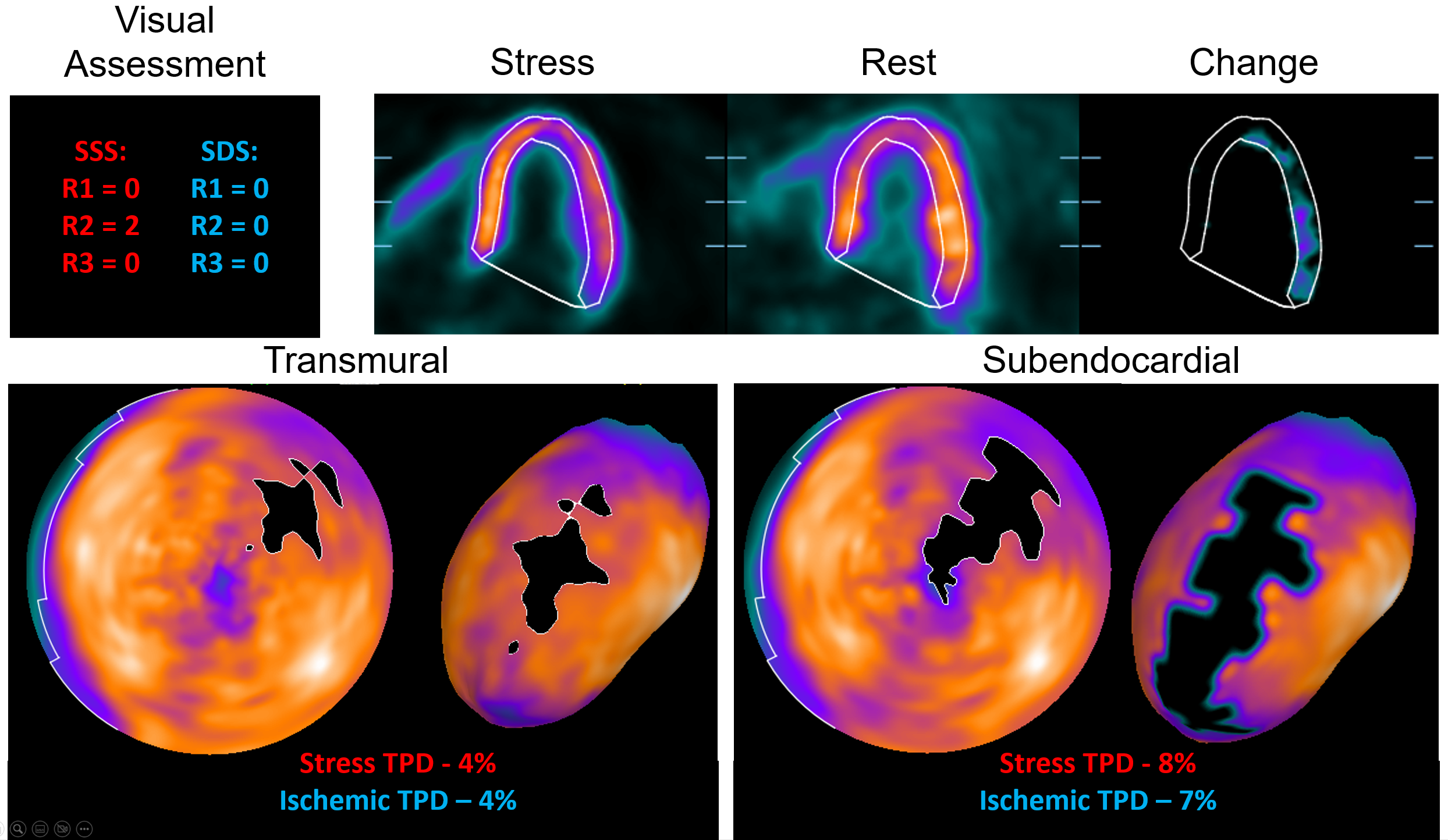
**

**Supplementary Figure 5**. **Case example**. Automatic quantification of transmural and subendocardial perfusion in an 83-year-old male patient with coronary artery disease, identified by invasive angiography as having 71% stenosis in the left circumflex artery. The top panel shows perfusion images in the vertical long axis (VLA) under stress (left), at rest (middle), and the difference between stress and rest (right). Transmural perfusion quantification indicated a stress total perfusion deficit (TPD) of 4% and ischemic TPD of 4% whereas subendocardial quantification indicated a stress TPD of 8% and ischemic TPD of 7%. The three readers’ (R1, R2, R3) summed stress scores (SSS) and summed difference scores (SDS) were considered to be normal.
